# Supplementary material for: VGEA: an RNA viral assembly toolkit
Source: PeerJ. 2021 Sep 6;9:e12129. doi: 10.7717/peerj.12129 (PMC8428259; doi:10.7717/peerj.12129)
Supplement: Supplemental Information 2 [file peerj-09-12129-s002.zip › Supplementary_File_2/Performance_Comparison_Assembly_Pipelines_Table_2/CV29/velvet/quast_results/results_2021_05_14_16_35_58/basic_stats/cumulative_plot.pdf]

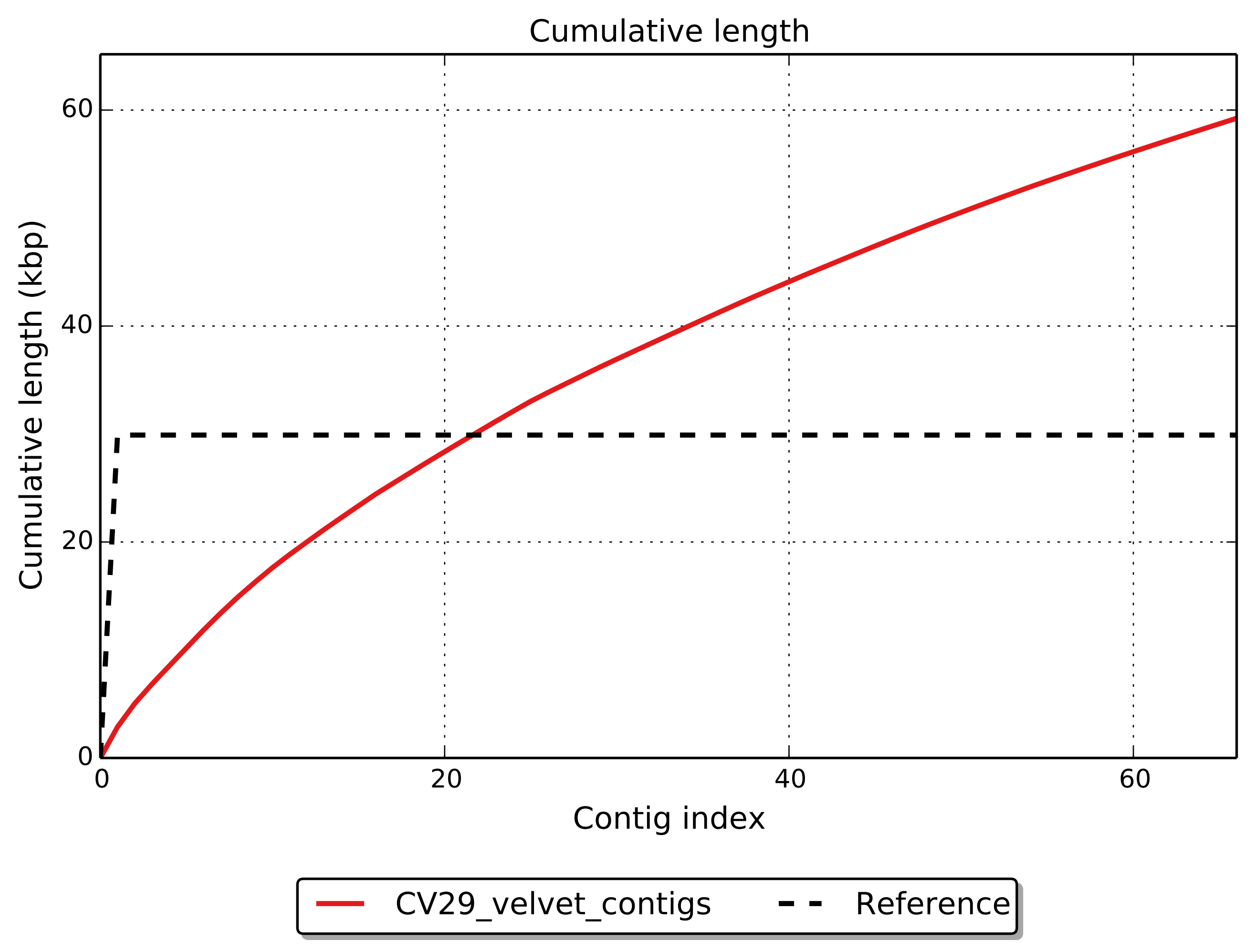

Cumulative length
Cumulative length (kbp)
0
20
40
60
0
20
40
60
Contig index
CV29_velvet_contigs
Reference
